# In vitro study of the embolic characteristics of imipenem/cilastatin particles

**DOI:** 10.1186/s42155-024-00441-x

**Published:** 2024-03-11

**Authors:** Hiroki Nakamura, Akira Yamamoto, Takeshi Fukunaga, Hiroyuki Watanabe, Kosuke Ito, Atushi Higaki, Akihiko Kanki, Yoshihiko Fukukura, Tsutomu Tamada

**Affiliations:** Departments of Radiology, Kawasaki Medical School, 577 , Matsushima, Kurashiki City, Okayama, Japan

**Keywords:** Imipenem/cilastatin, IPM/CS, Particle, Electron microscopy

## Abstract

**Background:**

Imipenem/cilastatin (IPM/CS) has long been administered intravenously as a carbapenem antibiotic. However, since this agent is poorly soluble in liquid, occasional reports have described its use as a short-acting, temporary embolic agent. The purpose of this study was to elucidate the characteristics of IPM/CS particles, which are thought to have pain-relieving effects against osteoarthritis-related pain, as an embolic agent.

**Methods:**

Three aspects of IPM/CS as an embolic agent were evaluated in vitro: particle size; particle shape; and change in particle size over time. For particle size, the long diameter was measured.

**Results:**

Mean particle size (*n*=244) was 29.2±12.0 µm (range, 1–60 µm). Shape (*n*=109) was round in 18.35%, elliptical in 11.93%, and polygonal in 69.72%, showing that most particles were polygonal. In observations of changes in particle size over time (*n*=9), particles had decreased to 75% of their original size at 82±10.7 min, 50% at 89.3±9.14 min, 25% at 91.3±8.74 min, complete dissolved at 91.8±9.02 min. A rapid shrinkage in diameter was seen in the final period.

**Conclusions:**

IPM/CS particles are ultrafine and the majority display a polygonal shape. This substance shows ultra-short embolic activity. This study revealed the characteristics of a substance that demonstrates an embolic effect not found in existing embolic materials.

## Background

Imipenem/cilastatin (IPM/CS) has long been administered intravenously as a carbapenem antibiotic. However, since this agent is poorly soluble in liquid, occasional reports have described its use as a short-acting, temporary embolic agent [1–3]. Some recent reports have also suggested that injection of IPM/CS has pain-relieving effects with respect to newly formed blood vessels accompanying chronic arthritis, as a novel treatment for the pain associated with osteoarthritis and other forms of chronic arthritis. This use of IPM/CS has also been considered effective against intractable pain that cannot be relieved with existing treatments, and has attracted attention as a novel method of treatment. The mechanisms underlying this pain-relieving effect are not currently understood, but since similar pain-relief is also obtained from embolization with existing small-diameter, permanent embolic agents, embolization of new blood vessels arising under proinflammatory conditions may produce pain-relieving effects [4, 5]. Moreover, since significantly fewer complications related to tissue ischemia arise with embolization using IPM/CS than with embolization using permanent embolic agents [5], the particle size and ultra-short temporary embolic effects of IPM/CS may produce selective embolic effects in new blood vessels arising under proinflammatory conditions, achieving effective pain-relief with few complications [2, 4, 5]. Although there have been several reports on the embolizing effects of IPM/CS [1–3], it is necessary to understand their more detailed characteristics.

The purpose of this study was to elucidate the characteristics of IPM/CS as an embolic agent in vitro under electron microscopy.

## Materials and methods

This study does not require ethics approval as it is an invitro study using no human or animal biological material. To elucidate the characteristics of IPM/CS(Merck&Co. Inc., Whitehouse Station, NJ, USA) as an embolic agent, the three characteristics of particle size, particle shape, and change in particle size over time were observed in vitro using a BZ-X electron microscope (Keyence Corporation, Osaka, Japan).

### Particle size

In measuring particle size, a non-ionic contrast agent (Iomeron350^®^; Bracco UK, Milano, Italy) was used same as when using IPM/CS as embolic agent which were observed under electron microscopy (20×) immediately after mixing in 2 mg of IPM/CS.

**Fig. 1:**
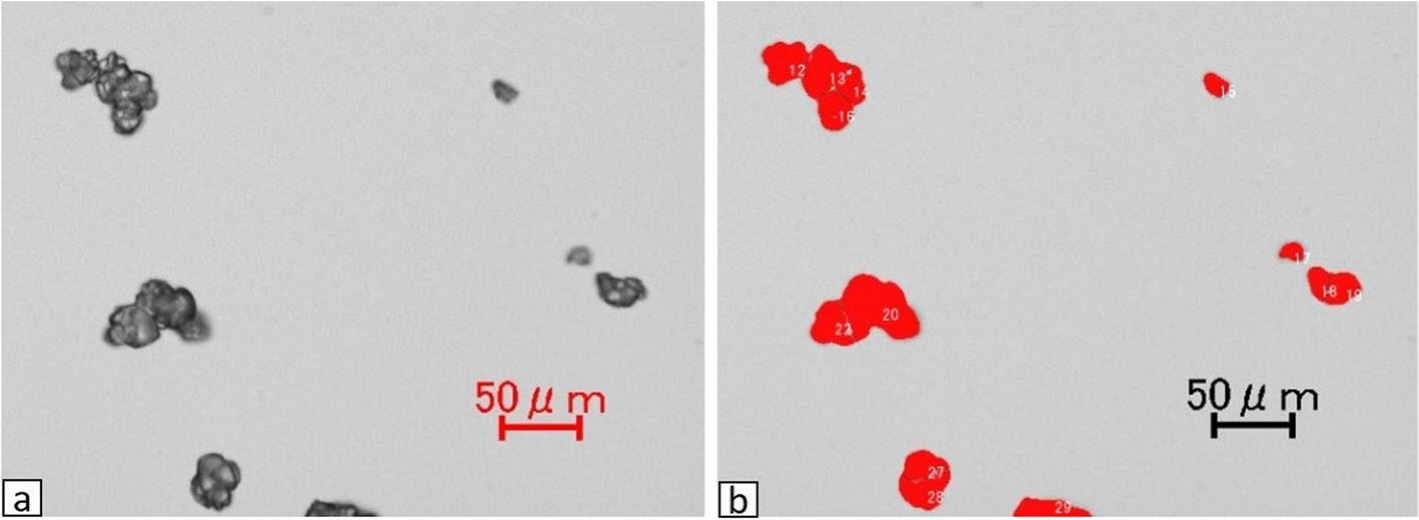
**a**) Image of IPM/CS particles immediately after dissolution in contrast agent.(x20) **b**) The long diameter of each particle was measured using the microcell counter in BZ-X Analyzer, an analysis application. Particles suspected of polymerization in visual judgments were excluded.(x20) The particles being measured are displayed in red

The entire field of view was observed first, then visual fields with little polymerization among particles were selectively imaged. Particles within a total of five visual fields were examined. The long diameters of particles were measured using the microcell counter of the BZ-X Analyzer analysis application (Keyence Corporation) (Fig. 1). When polymerization among particles was suspected from visual judgments those particles were excluded.

### Particle shape

First, 2 mg of IPM/CS was mixed into non-ionic contrast agent (Iomeron350^®^; Bracco UK) as the solvent. Images were immediately taken under electron microscopy (40×) and observed.

**Fig. 2:**
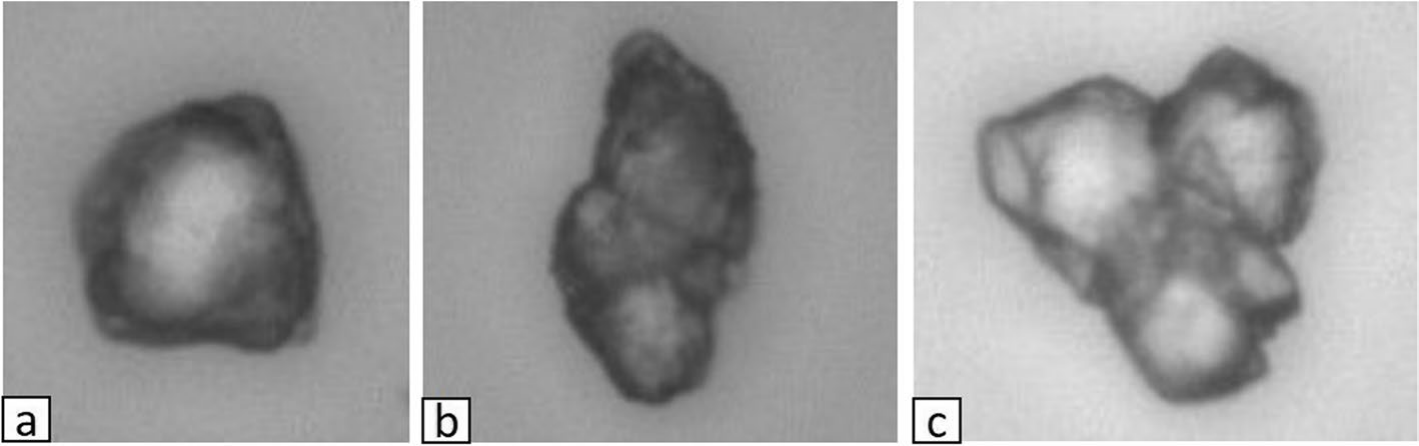
Particle shape is classified as round (**a**), elliptical (**b**), or polygonal (**c**). A polygonal shape is one with three of more angles ≤90°, with any other shapes defined as either round or elliptical. An elliptical shape is taken as one with a short axis/long axis <0.75, and a round shape as one with a short axis/long axis ≥0.75. **a** Round (long axis/short axis = 0.92) (40×). **b** Elliptical (long axis/short axis = 0.58) (40×). **c** Polygonal (40×)

Two radiologists performed these evaluations, visually categorizing shapes as round, elliptical, or polygonal (Fig. 2). The shape was defined as polygonal if angles ≤90° were seen for ≥3 vertices. All other particles were classified as either round or elliptical; particles for which the ratio of the short diameter to the long diameter was <0.75 were taken as elliptical; and particles for which the ratio of the short diameter to the long diameter was ≥0.75 were taken as round. For these visual judgments, particles showing suspected polymerization with other particles were excluded.

### Changes in particle size over time

First, 15 mg of IPM/CS was dissolved into 2 ml of physiological saline (0.9% NaCl saline solution) and observed under electron microscopy (20×) at room temperature. Time-lapse imaging (29 frames/min) was started from immediately after addition dissolution until all particles had disappeared. To avoid movement of the IPM/CS particles during the course of observation, a 12-well miniGPS^®^ Dish (Origio Japan KK, Kanagawa, Japan), which is a mortar-shaped dish for *in vitro* fertilization, was used as a receptacle for observation. The long diameter of particles within the imaging range that could be observed continuously from immediately after mixing until the particle had dissolved was measured every 2 min from immediately after mixing until the particle dissolved.

## Results

### Particle size

**Fig. 3:**
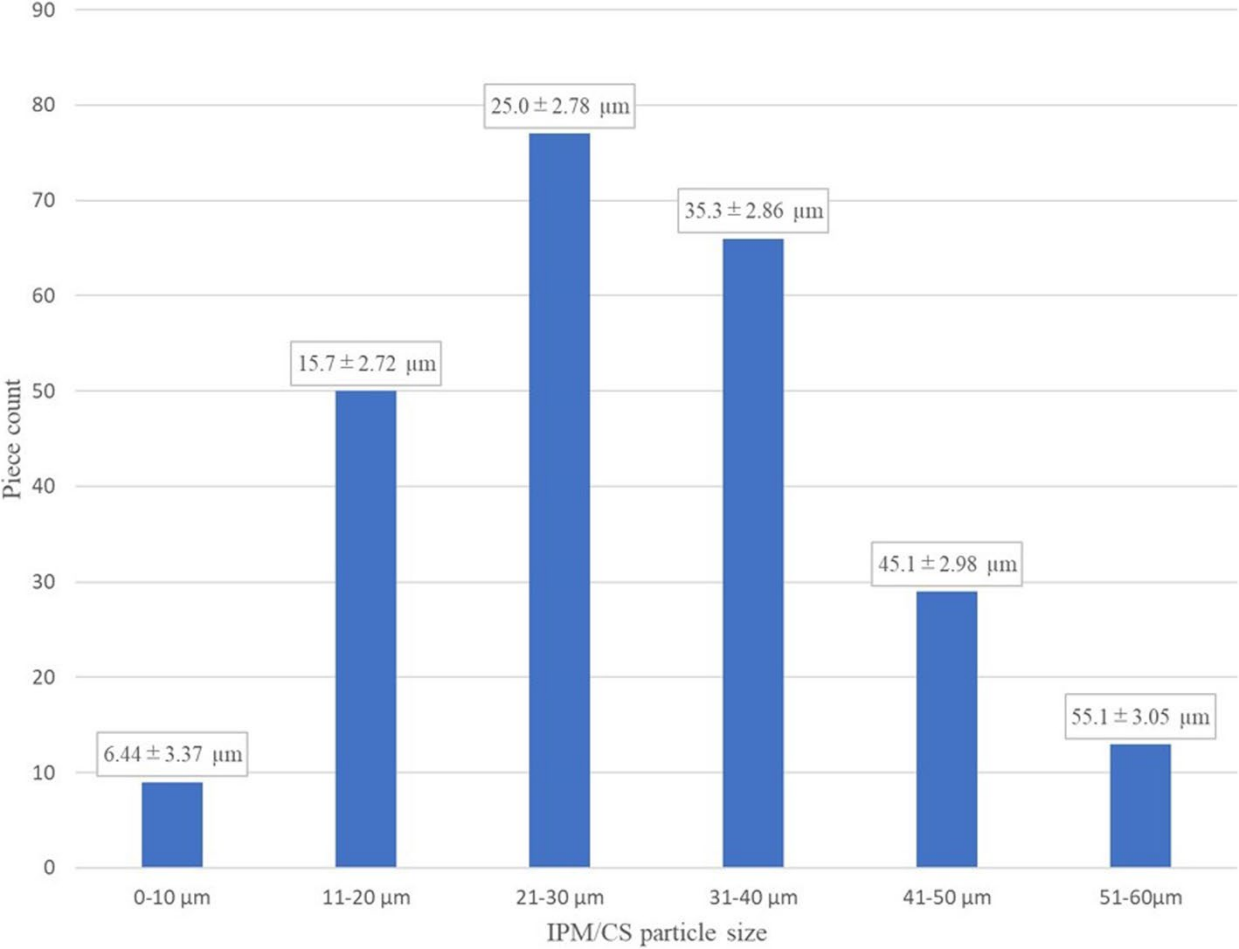
Distribution of long-axis lengths for IPM/CS particles

Measurements were made for 244 particles. Mean long diameter of particles was 29.2±12.0 µm (range, 1–60 µm). Particle size distribution was: 0–10 µm, 3.7% (9 of 244 particles); 11–20 µm, 20.5% (50 of 244 particles); 21–30 µm, 31.6% (77 of 244 particles); 31–40 µm, 27.0% (66 of 244 particles); 41–50 µm, 11.9% (29 of 244 particles); and 51–60 µm, 5.3% (13 of 244 particles). The most frequent size was 21–30 µm, and all particles showed a long diameter ≤60 µm (Fig. 3).

### Particle shape

**Fig. 4:**
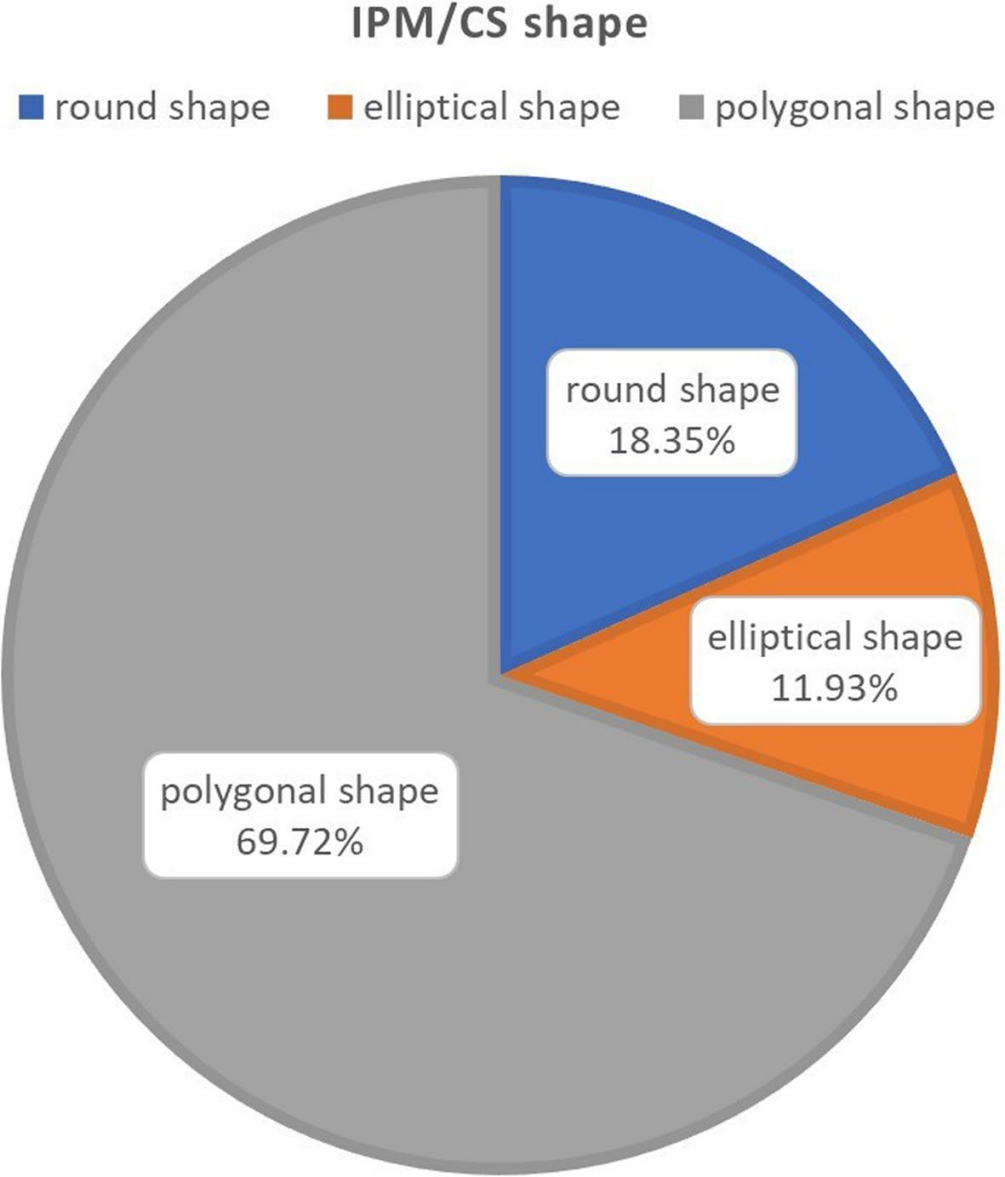
The shape is round in 18.35% of particles, elliptical in 11.93%, and polygonal in 69.72%. More than two-thirds present a polygonal shape

A total of 109 particles were categorized. The shape was round in 18.35% (20 of 109 particles), elliptical in 11.93% (13 of 109 particles), and polygonal in 69.72% (76 of 109 particles). The most common shape was polygonal (Fig. 4).

### Changes in particle size over time

Of the particles within the imaging range that could be observed continuously until dissolved, 9 particles (mean length, 32.9±5.74 µm; range, 25–42 µm) were measured after excluding particles that were extremely large or small.

**Fig. 5:**
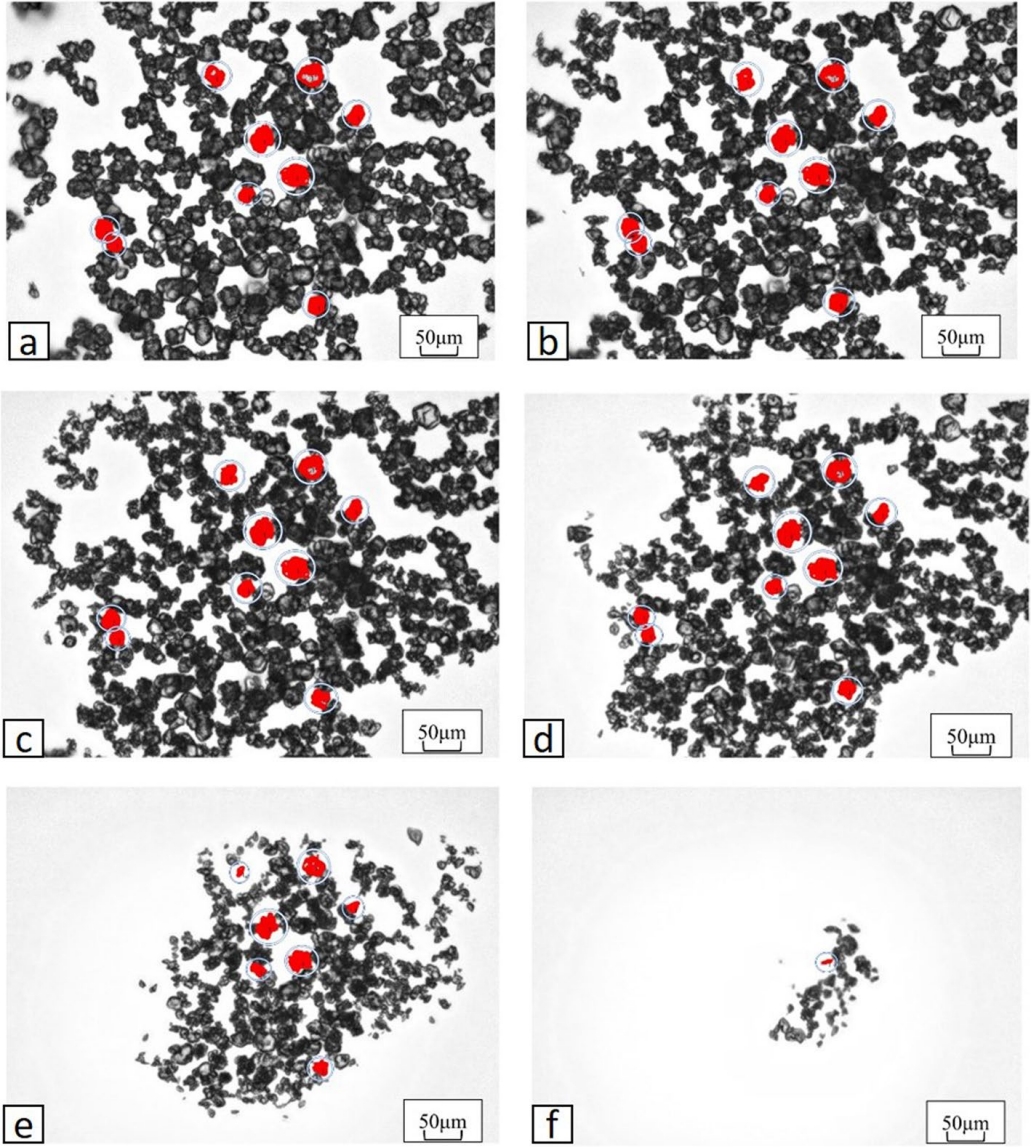
Changes over time until particle disappearance for the 9 observed particles under time-lapse imaging. **a** Immediately after dissolution (20×) The particles being measured are displayed in red. **b** After 20 min (20×). **c** After 40 min (20×). **d** After 60 min (20×). **e** After 80 min (20×). **f** After 100 min (20×)

**Fig. 6:**
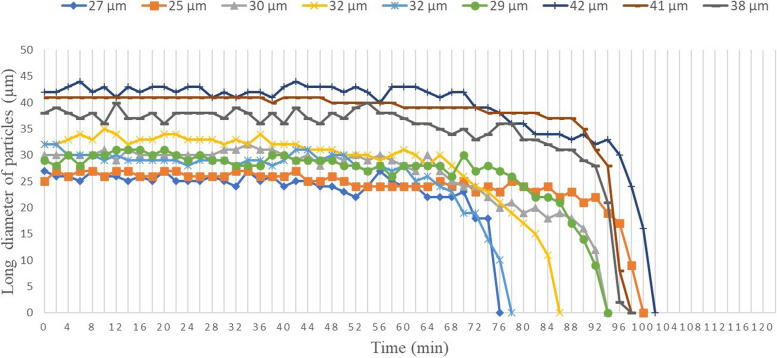
Changes over time in long diameter of the 9 observed particles until disappearance

Particles had decreased to 75% of their original size at 82±10.7 min, 50% at 89.3±9.14 min, 25% at 91.3±8.74 min, complete dissolved at 91.8±9.02 min (Fig. 5). Changes in particle size were not uniform; rather, rapid shrinkage was seen in the final period (Fig. 6).

## Discussion

IPM/CS comprises imipenem hydrate and cilastatin sodium. Imipenem hydrate is a white to pale yellow crystalline powder that is poorly soluble in water and slightly soluble in methanol. Cilastatin sodium is a white to pale yellowish powder that is readily soluble in water and in methanol [6].

In a report by Aihara, particle size in a turbid solution of 0.5 g of IPM/CS and 10 ml of contrast agent was consistently within the range of 30–60 µm, albeit with conspicuous irregularities [7]. These findings are not inconsistent with our results. In frozen sections immediately after administration of IPM/CS to animals, images showed embolism in blood vessels with diameters ≤60 µm, the same size as the particles [8].

Yamada et al. compared renal artery embolization with IPM/CS and Embozene^TM^ microspheres (40 μm) in rats [9]. No recanalization was seen with Embozene^TM^ microsphere embolization, whereas complete recanalization with IPM/CS was seen within 48 h. In histological evaluations, acute tubular necrosis (ATN) was seen with embolization using IPM/CS, predominantly in the renal cortex. This demonstrated the embolization effect of IPM/CS. ATN is thought to be seen with ischemia lasting 35–47 min [10, 11], and can occur even with the embolization time achieved by IPM/CS (91.8±9.02 min) in the present study, so these findings are not inconsistent with our results.

Okuno et al. also compared Embozene^TM^ microspheres (75 μm) with IPM/CS, this time in vascular embolization for osteoarthritis. They reported similar results in terms of pain relief, but transient changes in skin color were seen in four of the seven patients treated with Embozene^TM^ microspheres [2]. Hwang et al. reported treatment with Embospheres^®^ (40–120 μm) and IPM/CS for chronic shoulder and elbow pain, again finding transient changes in skin color for one of the four patients using Embospheres^®^ [4]. In a study by Casadaban et al., changes in skin color occurred in 63% of patients with Embozene^TM^ microspheres and persisted 1–3 months, whereas with IPM/CS this complication showed a frequency of only 2.5% and persisted for 3 weeks [5]. In those reports comparing IPM/CS with existing permanent embolic agents for chronic joint pain, the Embozene^TM^ (75 µm) and Embospheres^®^ (40–120 μm) used showed similar particle sizes to IPM/CS, and both groups exhibited pain-relieving effects. These findings suggest that ultrafine particles of several tens to hundreds of micrometers in diameter are effective for pain relief. This may be because the caliber of new blood vessels induced by chronic inflammation is small, and the embolic agents may reach all the way to these new vessels. At the same time, differences are seen with respect to duration of embolization with permanent embolic agents such as Embozene^TM^ microspheres and Embospheres^®^ as opposed to the ultra-short, temporary embolic agent IPM/CS. This difference may affect complications attributable to tissue ischemia. From these results, ultra-short, temporary embolic agents with an ultrafine particle size of several tens to hundreds of micrometers may have a characteristic of few complications in healthy tissue while producing pain-relieving effects. One cause of this result, although currently only hypothetical, may be that fragile new blood vessels arising under proinflammatory conditions are damaged by embolization more rapidly than normal blood vessels [12]. Moreover, the shape change characteristic of rapid shrinkage in the end stage, as shown in this study, may effectively and specifically damage new blood vessels caused by inflammation. Clarification of these issues is an important topic for future study. Embolization in osteoarthritis with embolic agents is still largely investigational with limited publication evidence.

In terms of shape, Embozene^TM^ microspheres, Embospheres^®^ and other permanent embolic agents are basically spherical, whereas most IPM/CS particles were polygonal. It has been reported that polyvinyl alcohol (PVA) particles, which has a polygonal shape, particles polymerize and has a significantly larger vessel were occluded than using microsphere particles (Embosphere^®^) of same size [13]. The IPM/CS observed in this study also exhibited various shapes, suggesting that it may be prone to polymerization. Ghelfi et al. reported that particles smaller than 100 µm seemed to be associated with more complications [14]. The polygonal shape of the particles, which leads to particle polymerization, may be preventing the complications related to tissue ischemia arise with embolization.

There are reports suggesting that although there is a tendency to think that the behavior of an embolic agent is the same regardless of the region where it is used, but it differs according to the joint, the vascular anastomoses, the collateral network, and the articular structures [15, 16]. It is considered important to reveal the characteristics of embolic materials under biological conditions in the future.

This study showed several limitations. First, the study was conducted at room temperature, and the results might be different under human body. However, the characteristics can be captured as it was conducted under a constant room temperature. Second, since the IPM/CS was deposited in a mortar-shaped dish, concentrations in solution may not have been uniform. However, since observations were made under fixed conditions, the effects on the results were likely small. Third, the effects of polymerization and aggregation of particles themselves during observations were unclear. Although such interactions affect particle size, particles suspected to have undergone polymerized according to visual judgments were excluded. This study was limited to in vitro data, and in vivo evaluations will also be necessary in the future.

## Conclusion

The present study observed particle size, shape, and changes in particle size over time for IPM/CS under electron microscopy. IPM/CS was found to provide ultra-short and temporary embolic activity with ultrafine, mostly polygonal particles. This study revealed the characteristics of a substance that demonstrates an embolic effect not found in existing embolic materials. In the future, it is necessary to clarify the characteristics of embolic materials under in vivo conditions.

## Data Availability

Not applicable.

## Abbreviations

IPM/CS: Imipenem/Cilastatin
ATN: Acute tubular necrosis
PVA: Polyvinyl alcohol
